# Exploring differences in perceptions of child feeding practices between parents and health care professionals: a qualitative study

**DOI:** 10.1186/s12889-021-11493-2

**Published:** 2021-07-23

**Authors:** Athira Rohit, Renae Kirkham, Leisa McCarthy, Valentina Puruntatameri, Louise Maple-Brown, Julie Brimblecombe

**Affiliations:** 1 Wellbeing and Preventable Chronic Diseases Division, Menzies School of Health Research, Charles Darwin University, Darwin, NT 0810 Australia; 2 Department of Endocrinology, Royal Darwin Hospital, Darwin, NT 0810 Australia; 3 Department of Nutrition, Dietetics and Food, Monash University, Melbourne, 3168 Australia

**Keywords:** Feeding practices, Aboriginal parenting, Aboriginal worldviews, Child nutrition

## Abstract

**Background:**

Evidence on child feeding practice is often based on the perspectives and experiences of parents and less that of health practitioners. In this study, we explored child feeding practice in Aboriginal communities in northern Australia from both the parents and health practitioners’ perspectives with the aim of informing nutrition improvement programs.

**Methods:**

Qualitative research methods were employed. Using semi-structured interviews, parents (*n* = 30) of children aged 2–5 years, and 29 service providers who were involved in the delivery of child health and nutrition programs in the same communities, were asked about child feeding attitudes and practices. Responses were analyzed through inductive and deductive analysis, recognizing that worldviews influence child feeding practices.

**Results:**

Sharing food was a central practice within families. Parents highly valued development of child independence in food behavior but were conflicted with the easy access to unhealthy food in their communities. This easy access to unhealthy food and inadequate food storage and kitchen facilities for some families were major challenges to achieving optimal diets for children identified by Aboriginal families and service providers. The responsive style of parenting described by parents was often misunderstood by service providers as sub-optimal parenting when viewed through a dominant western lens.

**Conclusions:**

Approaches to support healthy feeding practices and optimal child nutrition require health-enabling food environments. Along with a community-based Aboriginal health workforce, it is paramount that the non-Aboriginal workforce be supported to be reflective of the impact of worldview on their practice, to ensure a culturally safe environment for families where parenting styles are understood and appropriately supported.

**Supplementary Information:**

The online version contains supplementary material available at 10.1186/s12889-021-11493-2.

## Background

Gestation to 2 years of age is a unique window of opportunity for the development of child feeding practices that support optimal growth and development [1]. Child feeding practice is defined as the attitudes of and behaviours and techniques used by parents and/or caregivers, to influence a child’s food behavior, including the types and amounts of food consumed [2]. Understanding the modifiable factors influencing a child’s diet are important not only for parents and families, but also for health professionals interacting with children and families/parents, to help shape diet-related behaviour and optimize growth and development. Based on the amount and quality of parental control of a child’s food behavior (demandingness) and the degree to which parents are accepting and sensitive to their children’s emotional and developmental needs (responsiveness) [3], feeding practices are traditionally characterized as; i) authoritative (high demandingness and high responsiveness), ii) authoritarian (high demandingness and low responsiveness), iii) indulgent/permissive (low demandingness and high responsiveness) and, iv) uninvolved (low demandingness and low responsiveness) [4–6]. This typology is generally used to assess the association between feeding practices and child health outcomes. The authoritative style, among western populations (within Australia and other westernized populations), has been extensively studied and positive associations with child weight status and psychosocial development with this style of parenting have been demonstrated [5, 7, 8]. The findings of these studies however, may not be generalizable to non-western populations [6, 9, 10].

In Australia, western concepts of child feeding prevail in the health system and can influence the practice of health care practitioners and their notions of good parenting. Health care practice models based on western constructs of health may therefore not be effective in addressing the health needs of non-western populations in Australia [11]. This is particularly pertinent to Aboriginal and Torres Strait Islander families living in remote communities of Australia who due to the high burden of childhood illness experienced, interact frequently with health-care providers early in a child’s life [12–15]. An extensive body of literature exists on the importance of health care services aligning with Aboriginal and Torres Strait Islander cultural values and health views. The evidence calls for Aboriginal and Torres Strait Islander ways of knowing and doing to be included in health care systems and processes [16–23].

Health Care in remote Australia is delivered by Aboriginal and Torres Strait Islander health practitioners alongside non-Aboriginal health practitioners. In a study on what constitutes a ‘good’ remote area nurse from the perspective of Aboriginal people, Dunbar et al. [24] identified that cultural safety underpinned by respect, relationships and responsibility [25] was a fundamental characteristic of what Aboriginal staff looked for in a remote area nurse. Community members prioritized how the remote area nurse related to them and how in this relationship understanding of culture, history and the community was shown [24]. In an attempt to educate western-educated health professionals working with Aboriginal parents and families in remote Australian communities Kruske et al. [26] documented the experiences and beliefs of Aboriginal families as they cared for their children in the first year of life. They found significant differences in parenting behaviors and child rearing to that of non-Aboriginal Australian families and confirmed characteristics of child rearing previously reported [26–29]. Aboriginal parents valued the agency shown by their children and shared parenting responsibilities with the broader family. The children were active agents in determining their own needs and included in all aspects of community life. Without the Australian health system understanding and supporting this child-led parenting, Kruske et al. [26] posed that health care would fail parents and their children. For effective health care, Kruske et al. [26] concluded that health providers must engage with parents and families on the issue of child development in ways that respect and incorporate Aboriginal parenting frameworks and worldviews.

Limited existing literature on Aboriginal child rearing practices is based on the perspectives and experiences of that of parents and families [26]. There is scant literature on child feeding practice from the perspective of health practitioners. To build on the study by Kruske et al. [26], we aimed to explore existing child feeding practices from the perspective of Aboriginal parents, families and health care service providers in the community. Our purpose was to determine if differences in perspectives of child feeding may be reflected in differing worldviews.

## Methods

Qualitative research methods using semi-structured interviews were employed as we sought to provide new and rich insights in to participants’ experiences of child feeding practice and the factors within their ‘lifeworld’ that influence this [30]. We conducted this study from a social constructionist premise that the understanding of reality is contextually embedded and shaped by worldview and cannot be measured empirically [31]. A social constructionist perspective recognizes how the meanings and interpretations of ‘what is real’ are influenced by the social, cultural and political forces within an individual’s lifeworld. This also means that a researcher cannot “bracket-off” their ‘worldview’ from the process of data analysis and interpretation [32]. We used a semi-structured interview guide (Box 1) to gain deeper insights into participants experiences and factors influencing their perceptions around child feeding practice.

### Setting

The study occurred in the Northern Territory (NT) of Australia where in 2016, 25.5% of the 228,833 people in the NT were Aboriginal. Populated areas of the NT, including the capital (Darwin), are classified as remote and about one quarter of people in the NT live in communities that are classified as very remote (i.e., very restricted access to services) [33]. The NT is culturally and linguistically diverse, with English often spoken as a third or fourth language. Political and social structures established with European colonization of Aboriginal and Torres Strait Islander peoples underpin the socio-economic disadvantage and health inequities experienced by Aboriginal Australians. The median weekly personal income for people aged 15 years and over in every remote communities in 2016 was $525 compared to $1052 in Darwin [34]. Indigenous food systems, that Indigenous Peoples have deep knowledge of and have lived with for millennia, supplement imported foods. These foods are sourced through community stores and through other outlets providing grocery and or pre-prepared foods in very remote communities, and from supermarkets and other food outlets in Darwin and other urban centres in the Northern Territory. Food prices are high in very remote communities and some urban centres due to geographical remoteness from supply centres and high remote food retail business overheads [35]. Aboriginal and Torres Strait Islander children living in remote Australia compared to non-Aboriginal Australian children experience disproportionate rates of nutrition-related health conditions [36, 37]. This includes low birth weight, undernutrition, respiratory illnesses, ear and skin disease, anaemia and dental caries. Preventable chronic diseases are also emerging at a much younger age among the Aboriginal and Torres Strait Islander population compared to the non-Aboriginal population [12–15]. Most very remote communities and urban centres have a health service serviced by the government or an Aboriginal Community Controlled Health Organization. Early childhood learning centres also exist in these areas. These services employ Aboriginal and non-Aboriginal staff.

### Sampling and recruitment

Recruitment of parents/caregivers and health care practitioners occurred in two stages. In stage one we recruited a convenience sample of parents through the Pregnancy and Adverse Neonatal Diabetes Outcomes in Remote Australia (PANDORA) study. PANDORA is a prospective birth cohort study which examines birth outcomes among Aboriginal and non-Aboriginal women with and without diabetes in pregnancy and their babies [38]. Women recruited to the PANDORA study were those who attended antenatal clinics in hospital outreach services in Darwin and Alice Springs in the Northern Territory and consented to participate. Aboriginal participants with children between the ages of 2–5 years who participated in the follow-up Wave 1 and came from six specific locations in the NT (including Darwin, two urban centres and three very remote communities) were invited by PANDORA study staff (Aboriginal and non-Aboriginal women) to participate in our qualitative study between March and July 2016 (at the time of their participation in Wave 1). In stage two, we approached health care professionals and early childhood learning program coordinators in the same six locations and asked them to nominate key service providers with knowledge of child health and nutrition. Service providers (both Aboriginal and non-Aboriginal providers) were then invited to participate to obtain their perspectives on feeding practices in parallel to parent perspectives.

### Data collection

The interview guide (Additional file 1) informed by Australian literature on child feeding practice [39] was developed to explore participant’s experience of child feeding and contextual influences. A local community researcher was employed to assist with recruitment and data collection, including English translation where required, in two of the six study regions. Interviews were conducted by AR and the local community researcher at a mutually convenient time and place organized with the participant. Interviews were firstly conducted with parents and care-givers. Themes that emerged from these interviews were then presented to and discussed with service provider participants to probe their perspectives of child feeding behavior among the population they serviced.

### Ethics

The study was conducted in accordance with the Declaration of Helsinki and ethics approval was provided by the Human Research Ethics Committees of the Northern Territory Department of Health and Menzies School of Health Research (HREC 2015–2425) [40]. Prior to conducting interviews, the research team went through the study information sheet with participants and informed written consent was obtained.

### Researcher position

The research team comprised Aboriginal and non-Aboriginal Australian researchers. A social constructionist perspective recognizes that a researcher cannot rid themselves of their ‘lifeworlds’ and therefore needs to reflect on how these may influence study findings and potential biases or even misinterpretations of the data [31, 41]. AR, a non- Aboriginal researcher and lead author was raised with an Indian cultural background and attained undergraduate university qualifications in India and postgraduate qualifications in Australia. AR commenced work in the Aboriginal and Torres Strait Islander Peoples health context in 2015 and had been involved in Maternal and Child nutrition research for the past 5 years [29, 42, 43]. Her own cross-cultural experience has provided her with a clear view on how cultural contexts and social structures influence one’s values, experience and behaviour. AR approached this qualitative study with limited pre-conceived assumptions on child feeding practice of Aboriginal parents which limits researcher biased theories to some extent [44]. Authors (RK, LMB, LM and JB) contributed their extensive experience in conducting qualitative research (RK, LM, JB) in Aboriginal and Torres Strait Islander Peoples health (RK, LMB, LM, JB) and nutrition (LM, JB). Authors (AR, RK, LMB and JB) were cognizant of the potential for data misinterpretation being non-Aboriginal and worked with authors LM and VP who provided an Aboriginal lens to data interpretation. The local community researchers including author VP, assisted AR in the field in two locations, by establishing rapport with participants and reflecting with AR on the meaning of participant responses from their perspective.

### Data analysis

Data analysis occurred in two stages. First, parent interviews were transcribed by AR and uploaded into NVivo (Version 11) software for data management and coding. A set of codes were inductively derived from the data and refined through verification with a second researcher (RK). AR then coded all data. Coded data were summarized, and themes identified from summary reports. Second, service provider interviews were transcribed and deductively coded in NVivo based according to the themes that emerged from the parent interview analysis. RK and AR independently coded five randomly selected interviews and through discussion reached consensus on the coding approach. Central to data analysis was the recognition that a parent/care-giver’s child feeding practice is likely to be strongly influenced by worldview [41]. Authors AR and JB drew on their observations and experiences of child feeding practice in different cultural contexts and knowledge shared with them on child rearing from their work and relationships with Aboriginal experts and considered this in relation to the emerging themes and existing literature on child feeding practices. Authors LM and VP reviewed these interpretations.

## Results

Thirty parents (28 mothers and 2 fathers, mean age of 30 ± 7 years) with children aged 3 ± 1 year (14 girls and 16 boys) participated. Among the 29 service providers, 14 were Aboriginal and 26 were female. Service providers included 16 health care practitioners, 4 family support service providers, 6 early childhood learning program service providers, one Aboriginal Liaison officer and an Aboriginal Elder.

### Family and sharing food is central

Most participants lived with one or more extended family member/s (e.g., grandparents, uncles, aunties, nephews) and shared meals (either cooked at home or ready-made purchased from a shop). The mother or grandparents were identified by most as the primary influencers of their child’s diet and siblings and cousins also as significant influencers. While several parents indicated their child to have unhealthy food only sometimes, they also commented that these foods were wanted by their child when their parent, cousins or others were observed by the child to consume these.

*“We buy coke, just for me and my partner. Then he sees us and asks for it. We can’t say anything then” –* Parent participant.

School and child care settings were also reported to provide meals to the children in attendance. Food provision through these settings was seen by service providers to provide food security for some families. Service providers highlighted food insecurity in association with overcrowding (due to inadequate housing stock in some communities) as a common issue they had observed that prevented availability and access to food for some families at different times. Community childhood learning settings were said by service providers to provide opportunity for service providers to talk with families about food insecurity issues; rarely however did families raise such concerns. In contrast, several Aboriginal health service providers commented on the influence they were able to have on healthy eating habits in the community through their relationships with parents.

*“At lunch time [Mothers] get taken home with a baby food and usually a hot lunch for the mother. We do activities like play group, but the food is the main thing” –* Non-Aboriginal participant, Early learning program manager.

*“I work here* [early learning program] *and I will always talk to them* [parents] *a lot. If your kids are hungry at home you should come here, we’ve got good food here” –* Aboriginal participant, Health care provider.

### Families pleased with child’s appetite

The majority of the parents expressed they were “happy” with their child’s approach to food indicating that their child ate everything that they were provided. Many parents aspired for their child to be brought up with a healthy diet.

*“It is hard for me to think that I am the one who made him like that [overweight). I just want my son to eat the right food, makes me feel good”* – Parent participant*.*

Service providers commented that children were very comfortable to accept or ask for food from other family members. While most parents referred to preparing meals at home daily for their children, service providers had the view that meals were purchased as takeaway rather than home prepared due to the lack of cooking and/or storage facilities in some homes.

“*… like it is a lot easier to get the takeaway and buy a pre-prepared meal than buying vegetables and I mean families may not have frying pans or cooking utensils at home it is just convenience.”* Non-Aboriginal participant, Early learning educator.

### Highly valued child autonomy

Parents implied a high level of autonomy afforded to the child with respect to child feeding. Children generally were said to eat when they felt hungry, ate whatever they liked to eat, and were admired when able to demonstrate they were capable of feeding themselves. Parents praised their child’s demonstration of independence such as their ability to go to the kitchen and serve themselves when hungry or to go to the fridge and select something to eat for immediate and/or later consumption.

Most parents said that they were generally ‘easy’ (not strict) with their child’s behaviour and valued their child’s own decision on when, and what, and how much to eat. Only a few parents referred to having set meal times in their family. Several parents specified that their child could eat anything they chose even if it included unhealthy food items.

*“I am just going to be easy watching him eating. It makes me feel good. Like I am happy the way he is”* – Parent participant.

Parents however also commented on strategies they used to encourage healthy food consumption and/or restrict unhealthy food consumption, such as offering a different food to one that was not liked, hiding healthy food in other food items, hiding food from the child or talking to their children about healthy and unhealthy food. However, most parents indicated that even if they did try to create boundaries regarding consumption of unhealthy food items, sometimes they would give in.

*“Because every time I go to the shop, he always wants coke. And I am trying to stop him from drinking coke. He cries, he can cry whole day for coke. Mum I want coke, I want coke. I say, you can’t drink it. You can easily get sick. Sometimes he listens. Sometimes he doesn’t. He wants to fight with me. Sometimes I give in...”* – Parent participant.

Service providers also had observed that parents in general responded to a child’s hunger cues, tended to provide whatever food was asked for, and did not tend to use persuasion to influence a child’s food intake. On the other hand, they had also observed parents asking their child if they were hungry and talking to the child to encourage them to eat healthy food. Most non-Aboriginal service providers agreed that parents had an ‘easy’ parenting style and thought there was minimal emphasis on ‘disciplining’ their children. They described parenting as child-led and that parents never said ‘no’ to their child/ren.

*“I see it as more of a parenting issue not wanting to say no to a child where it is not helpful towards the child to make their own choices when they are 3 or 4 in what they eat, what they don’t eat. So, I see it as more of a parenting issue that parents don’t know how to say no to their children because they don’t want the reaction of saying no” –* Non-Aboriginal participant, Health care provider.

Aboriginal service providers thought that parents should say ‘no’ to their child/ren and that those that did were ‘strong’, but that not all showed this same behaviour to their child/ren.

*I see a lot of things happening with the parents and kids these days. Because kids are really crying for this junk food, but mum can’t give that “you don’t take this” they should give healthy foods. But kids go like mad and cry and what they (parents) do is they buy for that 1 day. “You shall start eating healthy food” that’s all they should talk. Some parents do that. But some don’t* – Aboriginal participant, Health care provider.

Service providers also commented on the younger generation “not learning the skills of their parents” and/or “forgetting their culture” by not being firmer with the children.

*No, it is not our culture. … when the child cries... they* [parents] *should say, no, you can’t have that. Why? Because it is not good for you. That’s what the parents should say to their kid. Talk them into it. Just talk to them. This is not, this is good for you. Get some fruit –* Aboriginal participant, Elder.

### Challenging food environment

Service providers commented that healthy foods were usually expensive and limited in variety in the community supermarket and that the unhealthy food items were often displayed at eye level and made very visible and attractive to children.

Service providers reported that tantrums at supermarkets were common when children didn’t get confectionery (lollies or chocolates). Most referred to having observed parents offering unhealthy food options in order to make their child stop complaining or crying about the food they desired. Several commented that a crying child is considered “shame business” (a situation that can cause embarrassment or disempowerment [45] as a child should not be distressed) for the parent and probably the reason for the parent to easily give in. Several parents commented on how they avoided taking their children to the supermarket to avoid ‘tantrums’.

*“I think it is hard. I do think the mothers give up sooner than later. It is hard, like I have seen children in the community, and I see them here (clinic) as well. They don’t like their child to be visibly distressed that is a shame job. So, they do give in easily because of the shame job*” – Non-Aboriginal participant, Health care provider.

*“But I don’t like [him] taking him to the shop. He might want lollies there. If I want to go to the shop, I will leave him with his aunty”* – Parent participant.

Service providers commented on the social (e.g., domestic violence, gambling) and health issues (e.g., sickness) that impacted some families’ abilities to provide healthy food for their children. Some non-Aboriginal service providers made judgements of the lifestyle of families in the communities and attributed issues with children’s diets to laziness; lack of time management, disciplining of children, and routine; and, questioned if parents actually followed their professional advice.

*“I think there are a lot of answers we want to hear. I think a lot of mothers talk about feeding that at breakfast, they really love the things that we like to hear, like Weet bix* – Non-Aboriginal participant, Health care provider.

### Strengthening child feeding practice

Service providers offered a wide range of strategies and advice on how they thought child feeding practices could be improved. Aboriginal service providers suggested strategies where Elders talk to young parents about the importance of providing the right food to their child/ren, involving children in activities like bush walks and other physical activities, families taking their children back to their homeland during holidays and, positive role modelling. Strategies offered by both Aboriginal and non-Aboriginal service providers included disciplining the child/ren and specifically saying ‘no’ to children, educating and reiterating to families the importance of a healthy diet and home cooked meals and, and providing money management training. Strategies to prevent tantrums at the supermarket were also offered.

*“They should take kids to the supermarket. Take the kids with them while going, make the kids busy with playing then the mother can do shopping. The kids can come with the mother but make sure the little kid behave himself. You can tell your kid to behave himself* – Aboriginal participant, Health care provider.

## Discussion

The views expressed by parents and service providers offer insight into the experience and perceptions of child feeding in the communities involved in this study. Service providers were aware of differences in child feeding behavior to that of Australian western practices and were aware of the incongruence between child-led parenting and an environment that undermined parent aspiration to guide healthy eating. Several non-Aboriginal service providers however viewed these differences as the problem. This demonstrates how different ‘life-worlds’ can influence perceptions of what is considered appropriate child feeding practice which may then influence how culturally safe parents feel when accessing health care.

Notable characteristics of child feeding practices on which both parents and service providers concurred include: parents being highly responsive and less demanding with their children, and, highly valuing their child’s autonomy in decision-making. These characteristics most closely resonate, from a western perspective, with a highly responsive and low demanding parenting style, where parents are generally warm and affectionate but reluctant to enforce ‘code of conduct’ with children [4, 5]. This responsive approach to child feeding along with an appreciation of child autonomy reflects an Aboriginal collectivist approach embedded in Aboriginal culture and worldviews [26, 28]. In a collectivist society, individuals are tightly integrated with each other and highly value the concept of ‘sharing’ which is quite contrasting to a western individualist society where loosely integrated individuals prioritize private matters [46]. Based on a collectivist view, a child born into an Aboriginal family is likely to learn the concept of ‘sharing’ early in life and to develop to be independent and confident within the extended nature of their family [47–50]. This enables the child to be autonomous in deciding the what, when and where in their interactions with parents and family. Kruske et al. [26] further describes that such practices reflect on how Aboriginal children are raised “strong”. On the other hand, children born to an individualist society are generally regarded as dependent and helpless and hence taught to follow routines and raised to follow instruction and be obedient. The worldview of an individualist society is likely to interpret the highly responsive and lower demanding behaviour of parents as potentially leading to negative outcomes for the child [50]. Hence, when viewed through this lens, such collectivist behaviour may be perceived as different and problematic, and therefore difficult to accept as also reported by Smith D et al. [51]. They observed different views on child growth between health professionals and community members in an Aboriginal community [51]. This was despite the recent literature indicating the benefits of responsive parenting as an appropriate way of child rearing [52].

This distinction in worldview is reflected in the responses elicited from the majority of non-Aboriginal service providers who interpreted their observations of parenting as an ‘undisciplined way of Aboriginal parenting’ and ‘parents unable to make a decision for their child’. This parenting approach was viewed as problematic particularly when it was seen to enable children to easily access and consume unhealthy food items. Aboriginal service providers tended to be less critical and where they had concerns, they provided strategies to strengthen child feeding practices. This included parents and family being positive role models, involving children in community related group activities, and parents guiding a child’s food choice. These strategies reflect the collectivist values and world views that promote Aboriginal children to bond with each other and their extended family [53]. Support for intergenerational knowledge transfer was another strategy identified by the Aboriginal service providers to strengthen child feeding practices. An ethnographic study that explored factors influencing food choice in a remote Aboriginal community observed similar findings regarding the importance placed on knowledge transfer within families. Aboriginal people in this ethnographic study expressed disconnect between their knowledge related to their long-established traditional diet and the limited knowledge they believed they had of the western diet [54]. The interest expressed by community members in empowering and guiding families to teach their children about the traditional food system as well as the western diet was highlighted in the same ethnographic study [54].

The existing food environment in the community and inadequate home infrastructure associated with food insecurity were major challenges to ‘positive’ child feeding practices identified by both participating families and service providers. Service providers referred to the high cost of healthier food items and easy accessibility of unhealthy food items they observed. Exposure to an unhealthy food environment is likely to clash with a ‘responsive/low demand’ style of parenting, particularly as young children may not have developed their cognitive capacity to discern between foods that are good or not good for their health and wellbeing [55]. Environmental cues such as colorful food packaging and branding in addition to pleasurable taste can interplay and interfere strongly with children’s ability to make healthy choices [55]. This vulnerability of children to the marketing of unhealthy foods is reflected in Australian government legislation such as the ‘Responsible Children’s Marketing Initiative’ [56]. Strategies put forward by the service providers to address these concerns included restriction of promotion of unhealthy food items in stores and in the community, enabling shops to have affordable and a wide variety of healthy items, and the provision of community level cooking classes. A systematic review of dietary interventions for Aboriginal and Torres Strait Islander Peoples [57] of Australia revealed that strategies including restriction of promotion of unhealthy food and drinks [58] and implementation of price discounts on fruits, vegetables, diet drinks and water [59] have shown positive purchasing patterns in remote Aboriginal communities. Such structural-ecological interventions that directly address the determinants of food insecurity and enable healthy food choices, rather than solely educating parents about healthy eating and feeding practices, are likely to positively contribute to strengthening child feeding practices [60–62].

Concerns expressed by the parents in relation to their child’s exposure and consumption of unhealthy food items were not known to service providers (majority health practitioners). This may be because parents often present at health services with acute concerns, thus providing limited health promotion opportunities, and/or because of limited trust to raise such issues with health personnel [63, 64]. Researchers involved in Aboriginal and Torres Strait Islander Peoples health research highlight the need for Australia’s health system to value its Aboriginal and Torres Strait Islander workforce and provide continuous care, that enables the establishment of meaningful and trusting relationships, and respects Aboriginal and Torres Strait Islander Peoples ways of knowing and doing [63, 64].

It is evident that the health practitioners in remote Aboriginal communities faces an enormous challenge when confronted with the day-to-day reality of child health issues that are diet-related but underpinned by structural issues outside of their realm of control and clinical training. This research shows a need for the health workforce and other service providers to be supported to confront the influence of worldview on practice. This is critical to providing meaningful services to families to strengthen and enhance child-feeding practices [65–67]. Anti-racist frameworks are emerging for organizations to support their employees to use self-reflection and confront white supremacy, and to put in place system-wide anti-racist change [68, 69]. Simultaneously, an empowered and strengthened Aboriginal health workforce within the Australian health system is critical to promote a culturally informed and appropriate work environment and the constructive exchange of concerns and education between Aboriginal families and health professionals and other service providers [16, 22, 70, 71].

The major strength of this study was the involvement of community researchers (one of whom (VP) contributed as a co-author) in two of the three very remote communities who created a safe space for Aboriginal parents to talk about their child’s diet and food behaviour with AR and helped with understanding child feeding from an Aboriginal worldview. In addition to strengthening participant engagement, they tuned into participant body language and semantics. For instance, the word ‘deadly’ may not mean dangerous or to be avoided, when used by respondents, but rather good and an accomplishment. The experience of child feeding shared from the perspective of the non-Aboriginal service providers interviewed, may be biased to those families who present to the health service for medical care. This clinical setting also may limit opportunity for meaningful discussion between clients and health practitioners on child feeding. The views discussed in this study are of those interviewed in the participating communities. We acknowledge the diversity of culture and social norms among Aboriginal and Torres Strait Islander Peoples and communities, and that therefore the findings may not reflect those of other parents, other service providers and/or other communities. However, the purpose of this qualitative research was to gain a richer insight into perspectives of child feeding practices and how these may be influenced by worldview. English was not the first language for some participants and though the community researchers assisted with language interpretation, it is likely to have influenced the depth of information that parents shared.

## Conclusions

This qualitative study provides insight to Aboriginal and non-Aboriginal worldviews as represented in experiences and perspectives of Aboriginal child feeding practice within several communities. Service providers unanimously stated the need for health-enabling food environments and opportunities for positive role-modelling in the community to support healthy feeding practices and improve child health and nutrition. The responsive/low demand parenting style of parents was misunderstood by some non-Aboriginal service providers as sub-optimal parenting when viewed through a dominant western individualist lens. Such disempowering views suggests the requirement for health services to support their staff who operate in complex and culturally diverse environments to practice culturally safe healthcare. Further, it highlights the importance of the Aboriginal health workforce who can promote Aboriginal ways of knowing and doing. Structures to support service providers confront the influence of worldview on practice are needed to provide culturally safe environments for families.

## Supplementary Information

**Additional file 1.**

## Data Availability

The datasets analysed during the current study are not publicly available due to identifiable information but ‘the Partnership’s Aboriginal and Torres Strait Islander advisory group will decide data sharing on reasonable request.

## Abbreviations

AR: Athira Rohit
HREC: Human Research Ethics Committees
JB: Julie Brimblecombe
LM: Leisa McCarthy
LMB: Louise Maple-Brown
NT: Northern Territory
PANDORA: Pregnancy and Adverse Neonatal Diabetes Outcomes in Remote Australia
RK: Renae Kirkham
VP: Valentina Puruntatameri

## References

[1] Arabena K, Ritte R, Panozzo S, Leah J, Rowley K. First 1000 days Australia: an Aboriginal and Torres Strait islander led early life intervention. Aborig Isl Health Work. 2016;40(Jan/Dec):21–2.

[2] Gevers DW, Kremers SP, de Vries NK, van Assema P (2014). Clarifying concepts of food parenting practices. A Delphi study with an application to snacking behavior. Appetite..

[3] Baumrind D (1967). Child care practices anteceding three patterns of preschool behavior. Genet Psychol Monogr.

[4] Baumrind D (1966). Effects of authoritative parental control on child behaviour. Child Dev.

[5] Maccoby E, Martin J, Mussen PHC L (1983). Socialization in the context of the family: Parentchild interaction. Handbook of child psychology: Socialization, personality, and social development. 4.

[6] Hughes SO, Power TG, Orlet Fisher J, Mueller S, Nicklas TA (2005). Revisiting a neglected construct: parenting styles in a child-feeding context. Appetite..

[7] Shloim N, Edelson LR, Martin N, Hetherington MM (2015). Parenting styles, feeding styles, feeding practices, and weight status in 4–12 year-old children: a systematic review of the literature. Front Psychol.

[8] Johnson R, Welk G, Saint-Maurice PF, Ihmels M (2012). Parenting styles and home obesogenic environments. Int J Environ Res Public Health.

[9] Tovar A, Hennessy E, Pirie A, Must A, Gute DM, Hyatt RR, Kamins C, Hughes SO, Boulos R, Sliwa S, Galvão H, Economos CD (2012). Feeding styles and child weight status among recent immigrant mother-child dyads. Int J Behav Nutr Phys Act.

[10] Olvera N, Power TG (2010). Brief report: parenting styles and obesity in Mexican American children: a longitudinal study. J Pediatr Psychol.

[11] Scrimgeour M, Dunbar T (2009). Cultural competence in Aboriginal education service delivery in Australia: Some lessons from the Aboriginal health service sector.

[12] Health AIo, Welfare (2018). Aboriginal and Torres Strait islander health performance framework (HPF) report 2017.

[13] Commonwealth of Australia. Closing the Gap Prime Minister’s Report. In: Department of the Prime Minister and Cabinet, editor. 2020.

[14] Azzopardi PS, Sawyer SM, Carlin JB, Degenhardt L, Brown N, Brown AD, Patton GC (2018). Health and wellbeing of indigenous adolescents in Australia: a systematic synthesis of population data. Lancet..

[15] Titmuss A, Davis EA, Brown A, Maple-Brown LJ. Emerging diabetes and metabolic conditions among Aboriginal and Torres Strait Islander young people. Med J Aust. 2019;210(3):111–3.e1. 10.5694/mja2.13002 30656687

[16] Smith K, Fatima Y, Knight S (2017). Are primary healthcare services culturally appropriate for Aboriginal people? Findings from a remote community. Aust J Prim Health..

[17] Harfield SG, Davy C, McArthur A, Munn Z, Brown A, Brown N (2018). Characteristics of Indigenous primary health care service delivery models: a systematic scoping review. Global Health.

[18] Lea T (2005). The work of forgetting: germs, aborigines and postcolonial expertise in the Northern Territory of Australia. Soc Sci Med.

[19] Durey A, Thompson SC (2012). Reducing the health disparities of Indigenous Australians: time to change focus. BMC Health Serv Res.

[20] Wilson AM, Magarey AM, Jones M, O'Donnell K, Kelly J (2015). Attitudes and characteristics of health professionals working in Aboriginal health. Rural Remote Health.

[21] Si D, Bailie RS, Togni SJ, DˈAbbs PHN, Robinson GW. (2006). Aboriginal health workers and diabetes care in remote community health centres: a mixed method analysis. Med J Aust.

[22] Clifford A, McCalman J, Bainbridge R, Tsey K (2015). Interventions to improve cultural competency in health care for indigenous peoples of Australia, New Zealand, Canada and the USA: a systematic review. Int J Qual Health Care.

[23] Mooney N, Bauman A, Westwood B, Kelaher B, Tibben B, Jalaludin B. A quantitative evaluation of Aboriginal cultural awareness training in an urban health service. Aborig Isl Health Work J. 2005;29:23–30.

[24] Dunbar T, Bourke L, Murakami-Gold L (2019). More than just numbers! Perceptions of remote area nurse staffing in Northern Territory government health clinics. Aust J Rural Health.

[25] Fleming T, Creedy DK, West R (2019). Evaluating awareness of cultural safety in the Australian midwifery workforce: a snapshot. Women Birth.

[26] Kruske S, Belton S, Wardaguga M, Narjic C (2012). Growing up our way: the first year of life in remote Aboriginal Australia. Qual Health Res.

[27] Little K, Sanson A, Zubrick SR. Do individual differences in temperament matter for indigenous children?: The structure and function of temperament in footprints in time. Fam Matters. 2012;91:92–105.

[28] Byers L, Kulitja S, Lowell A, Kruske S (2012). ‘Hear our stories’: child-rearing practices of a remote Australian Aboriginal community. Aust J Rural Health.

[29] Rohit A, Tonkin E, Maple-Brown L, Golley R, McCarthy L, Brimblecombe J (2019). Parent feeding practices in the Australian indigenous population within the context of non-indigenous Australians and indigenous populations in other high income countries – a scoping review. Adv Nutr.

[30] Moustakas C. Phenomenological research methods. SAGE publications; 1994. 10.4135/9781412995658.

[31] Patton MQ. Qualitative evaluation and research methods. SAGE publications; 1990.

[32] Bynum WEI, Artino ARJ, Uijtdehaage S, Webb AMB, Varpio L (2019). Sentinel emotional events: the nature, triggers, and effects of shame experiences in medical residents. Acad Med.

[33] Australian Bureau of Statistics. The Australian Statistical Geography Standard (ASGS) remoteness structure. Available from: https://www.abs.gov.au/websitedbs/d3310114.nsf/home/remoteness+structure. Accessed 17 May 2021.

[34] ABS (2016). 2016 Census QuickStats Northern Territory (STE), Indigenous Profile.

[35] NT Market Basket Survey 2019. Northern Territory Government. 2020. Available from: NT Market Basket Survey 2019 - Datasets - NTG Open Data Portal. Accessed 17 May 2021.

[36] Colles S, Maypilama E, Brimblecombe J (2014). Food, food choice and nutrition promotion in a remote Australian Aboriginal community. Aust J Prim Health.

[37] McCarthy L, Chang AB, Brimblecombe J (2018). Food security experiences of Aboriginal and Torres Strait islander families with young children in an urban setting: influencing factors and coping strategies. Int J Environ Res Public Health.

[38] Maple-Brown LJ, Brown A, Lee IL, Connors C, Oats J, McIntyre HD (2013). Pregnancy and neonatal diabetes outcomes in remote Australia (PANDORA) study. BMC Pregnancy Childbirth.

[39] Jansen E, Mallan KM, Nicholson JM, Daniels LA (2014). The feeding practices and structure questionnaire: construction and initial validation in a sample of Australian first-time mothers and their 2-year olds. Int J Behav Nutr Phys Act.

[40] NHMRC (2018). Road map 3: a strategic framework for improving Aboriginal and Torres Strait islander health through research.

[41] Smith LT (2012). Decolonizing methodologies: research and indigenous peoples: zed books.

[42] Rohit A, Brimblecombe J, O'Dea K, Tonkin E, Maypilama L, Maple-Brown L (2018). Development of a short-item diet quality questionnaire for indigenous mothers and their young children: the Menzies remote short-item dietary assessment tool. Aust J Rural Health.

[43] Tonkin E, Kennedy D, Golley R, Byrne R, Rohit A, Kearns T, et al. The Relative Validity of the Menzies Remote Short-Item Dietary Assessment Tool (MRSDAT) in Aboriginal Australian Children Aged 6−36 Months. Nutrients. 2018;10(5):590. 10.3390/nu10050590 PMC5986470 29748493

[44] Creswell JW (2013). Standards of validation and evaluation. Qualitative inquiry and research design: choosing among five approaches. 3rd.

[45] Vallance RJ (2001). A Cultural Bridge. Annual Meeting of the Australian Association for Research in Education; December 2–6; Fremantle, Australia.

[46] Hofstede G (1983). The cultural relativity of organizational practices and theories. J Int Bus Stud.

[47] Hamilton A (1981). Nature and nurture : Aboriginal child-rearing in north-central Arnhem Land. Australian Institute of Aboriginal S.

[48] Harrison L (1986). Diet and nutrition in a Tiwi community. A study of factors affecting the health status of under threes at Milikapiti, North Australia: PhD Thesis, Australian National University.

[49] Priest K, King S, Nangala I, Brown WN, Nangala M (2008). Warrki Jarrinjaku ‘working together everyone and listening’: growing together as leaders for Aboriginal children in remote Central Australia. Eur Early Child Educ Res J.

[50] Kearins J (1984). Child-rearing practices in Australia : variation with life-style.

[51] Smith D, Mununggurr L, Bamundurruwuy D, Edmond K (2003). How children grow: indigenous and health professional perceptions.

[52] Harbron J, Booley S, Najaar B, Day C (2013). Responsive feeding: establishing healthy eating behaviour early on in life. South Afr J Clin Nutr.

[53] Australian Institute of Family Studies. Strengths of Australian Aboriginal cultural practices in family life and child rearing. Child Family Community Australia. 2014; Paper No 25.

[54] Brimblecombe J, Maypilama E, Colles S, Scarlett M, Dhurrkay JG, Ritchie J, O’Dea K (2014). Factors influencing food choice in an Australian Aboriginal community. Qual Health Res.

[55] Elliott C, Brierley M (2012). Healthy choice?: exploring how children evaluate the healthfulness of packaged foods. Can J Public Health.

[56] Australian Food and Grocery Council (2018). Responsible CHILDREN'S marketing INITIATIVE.

[57] Gwynn J, Sim K, Searle T, Senior A, Lee A, Brimblecombe J (2019). Effect of nutrition interventions on diet-related and health outcomes of Aboriginal and Torres Strait islander Australians: a systematic review. BMJ Open.

[58] Brimblecombe J, McMahon E, Ferguson M, De Silva K, Peeters A, Miles E (2020). Effect of restricted retail merchandising of discretionary food and beverages on population diet: a pragmatic randomised controlled trial. Lancet Planet Health.

[59] Brimblecombe J, Ferguson M, Chatfield MD, Liberato SC, Gunther A, Ball K, Moodie M, Miles E, Magnus A, Mhurchu CN, Leach AJ, Bailie R, SHOP@RIC research collaborative (2017). Effect of a price discount and consumer education strategy on food and beverage purchases in remote indigenous Australia: a stepped-wedge randomised controlled trial. Lancet Public Health.

[60] Green L, Kreuter M (2005). Health program planning: an educational and ecological approach: McGraw-hill education.

[61] Brimblecombe J, Bailie R, van den Boogaard C, Wood B, Liberato SC, Ferguson M, Coveney J, Jaenke R, Ritchie J (2017). Feasibility of a novel participatory multi-sector continuous improvement approach to enhance food security in remote indigenous Australian communities. SSM Population Health.

[62] Shaw G (2002). An Ethnographic Exploration of the Development in Child Rearing Style among the Ngaanyatjarra People from the Pre-Contact Era to the Present: Masters Thesis, University of New South Wales.

[63] Kildea S, Tracy S, Sherwood J, Magick-Dennis F, Barclay L (2016). Improving maternity services for indigenous women in Australia: moving from policy to practice. Med J Aust.

[64] Dunbar T (2011). Aboriginal People's experiences of health and family Services in the Northern Territory. Int J Crit Indigenous Stud.

[65] Reibel T, Walker R (2010). Antenatal services for Aboriginal women: the relevance of cultural competence. Qual Prim Care.

[66] Bourke S, Wright A, Guthrie J, Russell L, Dunbar T, Lovett R (2018). Evidence review of indigenous culture for health and wellbeing. Int J Health Wellness Soc.

[67] Lowitja Institute (2014). Cultural competence of mainstream health services and systems roundtable.

[68] Came H, Griffith D (2018). Tackling racism as a “wicked” public health problem: enabling allies in anti-racism praxis. Soc Sci Med.

[69] McGuire-Adams T. Settler allies are made, not self-proclaimed: unsettling conversations for non-indigenous researchers and educators involved in indigenous health. Health Educ J. 2021:001789692110092. Settler allies are made, not self-proclaimed: Unsettling conversations for non-Indigenous researchers and educators involved in Indigenous health - Tricia McGuire-Adams, 2021 (sagepub.com).

[70] Jongen C, McCalman J, Bainbridge R (2018). Health workforce cultural competency interventions: a systematic scoping review. BMC Health Serv Res.

[71] Gwynne K, Lincoln M (2017). Developing the rural health workforce to improve Australian Aboriginal and Torres Strait islander health outcomes: a systematic review. Aust Health Rev.

