# Supplementary material for: Exploring differences in perceptions of child feeding practices between parents and health care professionals: a qualitative study
Source: BMC Public Health. 2021 Jul 23;21:1449. doi: 10.1186/s12889-021-11493-2 (PMC8299622; doi:10.1186/s12889-021-11493-2)
Supplement: Supplementary file 1 — Additional file 1. [file 12889_2021_11493_MOESM1_ESM.docx]

**Interview guide**

1. Start with child’s usual activities including diet as soon as he/she gets up in the morning
   1. Foods that are encouraged or restricted to eat
   2. Techniques used to encourage or restrict
   3. Reasons for encouragement or restriction
2. To what extent do you influence what your child eats?
   1. Other settings that could influence child’s diet (child care, family members etc.)
   2. Parents thoughts about the influence of such settings on their child’s food behaviour
3. What do you do when your child is hungry?
   1. About hunger cues
   2. Strict or easy going - parenting
   3. Response to child tantrums in relation to food and saying ‘no’ to the child (food restriction)

Appendix 1 Interview guide for parent participants
